# Marginal Players? The Third Sector and Employability Services for Migrants, Refugees and Asylum Seekers in the UK

**DOI:** 10.1007/s11266-020-00306-6

**Published:** 2021-01-04

**Authors:** Francesca Calò, Tom Montgomery, Simone Baglioni

**Affiliations:** 1 PuLSE, The Open University, Milton Keynes, UK; 2 Yunus Centre for Social Business and Health, Glasgow Caledonian University, Glasgow, Scotland; 3 Department of Management, University of Parma, Parma, Italy

**Keywords:** Labour integration, Migration, Third sector, Marketization

## Abstract

Literature in the field of employability and the third sector has focused upon the impact of marketisation on third sector providers, elaborating how commissioning processes have led to a contraction of (smaller) third sector organisations (TSOs) and an expansion of larger private sector bodies. Extant research does not however explore the role of third sector organisations in the employability of migrants, refugees and asylum seekers. Therefore, our paper explores this gap by adopting a qualitative approach via a total of 36 interviews involving migrants, refugees, asylum seekers and managers of third sector organisations, alongside a categorisation of TSOs. Our findings reveal that TSOs are the primary (and for asylum seekers perhaps the only) providers of integration support services and training or education services. We found that only a limited number of organisations provide formal employability services or skills development services which seem to be only residual in terms of the range of activities that TSOs can organise. Thus, perhaps the main function that TSOs perform that enables integration into the UK labour market is providing a safe and trusted environment that people can use to increase their confidence, improve their well-being, broaden their social circle, learn the language or increase their work experience.

## Introduction

The issue of migration has been a contentious issue policy and public terrain in the UK for decades (Geddens and Scholten 2016). In the contemporary setting, it has been mobilised as a rhetorical rallying point for those seeking to garner popular support for the leave campaign during the Brexit referendum. Much of the anti-EU rhetoric was channelled through an anti-migrant discourse which oscillated between portrayals of foreigners as exploiting the country’s welfare system on the one hand and stealing jobs on the other hand (Dennison and Geddes 2018). However, the political turbulence that has been strategically generated in the name of migration has not prevented newcomers from building their lives in the country, nor has it managed to sweep away the UK’s history as a society that has migration woven into its fabric (Modood 2012; Rattansi 2011). Hence, in the interstices of such a contentious debate there has been space for the political elite to recalibrate the narrative of migration to better fit the history of the country and the demands of the labour market. Such a refocusing of the political narrative of migration (through a decade of conservative government) has borrowed from a mainstream policy discourse of ‘deservingness’ and therefore has developed alongside an assumption that migrants must prove their ‘worth’ to their host country (Sales 2002). Much of this deservingness is framed around their capacity to contribute to economic growth, and thus being economically self-reliant.

However, there are various challenges newcomers must overcome to be able to access the labour market and become self-reliant: they must learn or even master the language in a sufficient manner, they must possess the legal status that allows them to work, a complex task which requires navigating the bureaucracy of the UK Home Office, and ultimately they must find someone who provides an opportunity for employment, an employer who is persuaded about their suitability for that specific job (Anderson 2010; Martín et al. 2016; Mulvey 2018). To successfully progress through such steps newcomers rely either upon the support of relatives and acquaintances, as many ‘native’ citizens would do, but they can also rely on a set of organisations and businesses that provide support to migrants at their different stages of progress and across a spectrum of needs. In fact, as in other social policy domains, the UK has in the field of labour and migration created a market area where a range of organisations operate to provide services on behalf of the state and its devolved, regional and local units of government. Similarly, as has been the case for other social policy areas, those bodies operating in this field found themselves navigating a context where despite being delegated much responsibility and expectation via policy paradigms such as the Big Society (Kisby 2010), they have been confronted by funding cuts following the post-2008 austerity policies which have strongly impacted their capacities (Kendall et al. 2018).

Among these organisations are those emanating from the so-called third sector referring to those organisational bodies that emerge in the spaces between the state and society, and that are often motivated by an altruistic esprit of helping others (while generating economic profits or not) (Salamon et al. 2003).

It is through the prism of civil society mobilisation in the field of labour that we focus in this article on the role that the third sector is called to play in the post-Big Society, post-austerity UK context, while appreciating the features of migration in the country. Despite the increasing importance of third sector organisations (TSOs) in delivering services, little existing research has focused on the role of these organisations in relation to migrants, refugees and asylum seekers. Even fewer scholars have explored in detail how third sector organisations contribute to the employability of migrants, refugees and asylum seekers in the UK. Our aim in this paper is to answer the research question: what is the importance of third sector organisations in favouring (or not) the employability of migrants, refugees and asylum seekers in the UK?

This article proceeds as follows. Firstly, we offer a brief account of the role of third sector organisations in the policy evolution of the UK, focusing on policies developed in relation to third sector organisations. Second, an overview of previous academic studies that explore the contribution of third sector organisations in the employability of migrants, refugees and asylum seekers is provided. Third, in the methodology section a description of the methods used for our study is elaborated. Fourth, in the findings section we analyse the role of TSOs in the integration of migrants, refugees and asylum seekers into the labour market. We then explore the relationship between TSOs and the public sector. In the final section we discuss 3 key considerations that must be drawn from our findings in terms of how TSOs contribute towards the labour market integration of migrants, refugees and asylum seekers and how this contribution interacts with the social, economic and political environment in the UK.

## Third Sector Organisations in the UK

Third sector organisations, understood as formal or informal groups which have some structure and regularity in their operations (Salamon et al. 2003), have performed a key role in the implementation of UK policy since the end of the 1970s. In 1979 the election of a Conservative UK Government led by Margaret Thatcher advanced “New Right” policies which established the subsidiary principle as a cornerstone of its idea of public policy-making, opening the way for the private sector to become the most relevant provider of what used to be ‘public services’ (Gamble 1994).

Similar policy trajectories were continued by the New Labour Government elected in 1997 and subsequently also by the Conservative-led Coalition Government which came to power in 2010 (Grand 1991; Haugh and Kitson 2007; Alcock 2016). During the New Labour Government, the state was conceptualised as an ‘enabler’ in promoting civic activism and engaging with civil society organisations to address societal needs (Carmel and Harlock 2008; Haugh and Kitson 2007), while at the same time, civil society organisations were promoted to foster community development and renewal (Johnson 1999). Third sector organisations were considered by many policy-makers to be better embedded in the community and thus better able to understand specific societal needs than many state actors (Haugh and Kitson 2007; Nicholls and Teasdale 2017). In the 2010 UK General Election, one central plank of the Conservative Party manifesto was that of the “Big Society” (Kisby 2010). A greater level of voluntarism, including paving the way for charities, private enterprises and social enterprises to be much more involved in the running of public services was subsequently encouraged, at least rhetorically, by the Coalition Government (Montgomery and Baglioni 2018).

The brief analysis of the historical role of TSOs in the UK offered here has traced a common commitment across different governments to expand the functions of these organisations and to support them in providing public services, treating them as a substitute to, or as a replacement for, existing (public) providers in a competitive market (Calò et al. 2018; Sepulveda 2015). This can also be identified in the provision of employability services. For example, literature in the field of employability and the third sector has focused upon the impact of marketisation on third sector providers (Zimmermann et al. 2014), elaborating how commissioning processes have led to a contraction of (smaller) TSOs and an expansion of larger private sector bodies (Egdell et al. 2016). These organisations have also been required to tailor their services and activities to meet the needs of public funders and this has generated challenges to provide added value such as service innovation and community engagement (Lindsay et al. 2014). The implementation of austerity policies in the UK following the 2008 crisis and the subsequent cuts to social policy programmes which have been well evidenced in extant studies (Lowndes and Pratchett 2012; Milbourne and Cushman 2015; Montgomery and Baglioni 2018) have been particularly acute in those local levels of government that TSOs often engage in partnership (Lowndes and McCaughie 2013). Are the dynamics above described true also in a politically contested field such as migration and in particular for those TSOs which operate in the specific area of labour market integration?

## TSOs, Migrants, Refugees and Labour Market Integration

The third sector which deals with migrants and refugees within the UK has been under-researched and under-theorised within academia in recent years (Mayblin and James 2019).

On the more general topic of migration and civil society, recent literature has focused on the conceptualisation of which organisations might be included under the heading of Black and Asian Minorities Ethnic organisations (BAME), on discussing the presence of a distinctive sector (McCabe and Phillimore 2017) and exploring the reason behind the lack of development of these TSOs (Craig 2011; McCabe and Phillimore 2017, Ware 2017). Third sector organisations which work with refugees have been defined as often small, local, volunteer run organisation which often fill gaps of public services (Phillimore and McCabe 2010). This is particularly true for asylum seekers and particularly refused one which can only access to third sector organisations to fill some of their needs (Mayblin and James 2019).

Literature has also discussed what was considered the poor levels of government support offered to refugee community organisations (Phillimore and Goodson 2010) and the impact of austerity on the BAME sector (Tilki et al. 2015; Ware 2017). For example, researchers have explored that funding opportunities for non-profit organisations dealing with BAME communities and in particular asylum seekers and refugees have been reduced across time (Mayblin and James 2019) and few community organisations operating for these beneficiaries have been in a position to bid for contracts due to their low annual turnover (Ware 2017). Scholars have also pointed to accommodation contracts previously awarded to local authorities and third sector organisations transferred to private security companies (e.g. COMPASS). Only one third sector organisation (Migrant Help) has been contracted by the central government to provide advice to migrants, refugees and asylum seekers. At the same time cuts to the budgets of the Refugee Council and Refugee Action led to a decline in essential employment and integration services (Terry 2017). Only in few contexts, such as the Scottish one, an orientation towards co-management and co-governance of services involving third sector organisation was identified (Strokosch and Osborne 2016). However, Strokosch and Osborne (2016) also discussed that other factors such as government policy and geography were among the fundamental variables to facilitate both integration and inter-organisational relationships. Hence, existing studies have revealed that across the years, policies have on balance shifted resources more towards the private sector oriented end of the spectrum than towards those grass-roots, civil society organisations (Lowndes and Pratchett 2012; Milbourne and Cushman 2015; Montgomery and Baglioni 2018). Hence, the logic of privatisation and marketisation of what used to be public social services has progressed since the Thatcher era into the contemporary service delivery aimed at migrants and refugees.

While, a substantial amount of studies have researched the basic services for migrants provided by TSOs, particularly in the first days and months of arrival in the receiving country (Garkisch et al. 2017), few have explored the relationship between TSOs, migrants/refugees/asylum seekers and the labour market and even less attention has been paid to these relationships in the UK context. According to a recent systematic review, research has focused on job training, the direct hiring of migrants, subsidised programmes and support with work permits and work contracts (Garkisch et al. 2017). However, few of these studies were conducted in the UK context. For example, Shutes (2011) highlighted how the welfare-to-work policy might conflict with an ethos to assist those refugees who are among the hardest to reach or to encourage better access to a range of matched skilled jobs. Some research has also been conducted on those campaigns to address issues of solidarity, provide legal services and promote voluntary work (Sales 2002). Meer et al. (2019) highlighted that third sector organisations in Scotland have sought to engage private sector employers through successful brokering programmes. However, scarce funding has limited the success and the capacity of these initiatives. Recently, De Jong (2019) discussed that TSOs provides employability opportunities for highly educated refugees thanks to the access through volunteering and the recognition of “refugeeness” as a form of capital. The risk of involving them in “hidden, devalued and unremunerated emotional labour” (De Jong 2019, p. 335) was also highlighted.

No existing research has focused exclusively on third sector organisations employability services for migrants, refugees and asylum seekers in the UK. Employability in our study includes all those activities which are directly related to employment, such as for example employment programmes, education and language training, skills development and policy advocacy for labour market integration.

Therefore, there is a gap for robust analysis to identify and understand how TSO organisations favour or not the employment of migrants, refugees and asylum seekers; findings from these analyses, which offer different perspectives, may well have important implications for policy development and best practice as we discuss in our conclusions.

## Methodology

In our paper, we opted for a mixed methods research approach, which included the collation and descriptive analysis of secondary data concerning third sector organisations to provide an overview and categorisation and qualitative semi-structured interviews to help in understanding the role of civil society organisations in the field.

### Categorisation of TSOs

Our overview and categorization of TSOs included organisations which provide activities that are related to employability of migrants, refugees and asylum seekers.^1^ An approach which brings together data from the Charity Commission, Scotland Charity Regulator and the Charity Commission for Northern Ireland was designed. The 3 databases were used to identify all registered organisations who support migrants (including BAME communities), refugees and asylum seekers. To meet the inclusion criteria organisations will have used the word “asylum”, “refugee”, “migrant”, “BME/BAME” in their activities description in the database. Each charity website and/or Facebook page were reviewed and only those organisations which provide activities related to employability were finally included. Those organisations which do not have a website or Facebook presence were excluded with the assumption that they were currently inactive. The dataset includes details on the year of foundation, activities, location and client groups. The dataset was analysed using Excel to provide an overview of the characteristics and activities of third sector organisations involved in the labour market integration of migrants, refugees and asylum seekers.

### Qualitative Interviews

During our data collection, a sampling strategy that sought to ensure the inclusion of a variety of perspectives was pursued. This was reflected in the sample of interviewees. A total of 36 interviews and one focus group were conducted; 18 interviews involved managers of third sector organisations providing different activities related to the employability of migrants, refugees and asylum seekers (see Table 1 for more details).

**Table 1:** Interviews with TSOs representatives

Interviews TSOs representatives	Function/Role	Type
Interview TSO 1	Founder	Community-based organisation
Interview TSO 2	Coordinator	NGO
Interview TSO 3	Founder	Social enterprise
Interview TSO 4	Founder and managing Director	Social enterprise
Interview TSO 5	Founder and director	Social enterprise
Interview TSO 6	Founder	Community-based organisation
Interview TSO 7	Coordinator of a specific project	NGO
Interview TSO 8	Volunteer coordinator and senior case worker	NGO
Interview TSO 9	Evaluation officer	NGO
Interview TSO 10	Coordinator of a specific project	NGO
Interview TSO 11	Coordinator of a specific project	Community-based organisation
Interview TSO 12	Deputy director	NGO
Interview TSO 13	Director	NGO
Interview TSO 14	Employment coordinator	NGO
Interview TSO 15	Chair	NGO
Interview TSO 16	Director	NGO
Interview TSO 17	Policy officer	NGO
Interview TSO 18	Manager	NGO

In tandem with our sampling of the managers of TSOs, we also conducted a total of 18 interviews and one focus group involving migrants, refugees and asylum seekers (see Table 2 for more details).

**Table 2:** Interviews with migrants, refugees and asylum seekers

Pseudonym of interviewee *	Date of interview	Age	Gender	Family status	Country of origin	Migration year	Education (primary, secondary, tertiary)	Current occupation in host country	Occupation in country of origin	Languages the individual speaks
Interview 1	02/10/2018	Mid 30 s	M	Single	India	2011	Tertiary	IT—Insurance Company	Working with IT	English, Indi
Interview 2	05/10/2018	Mid 30 s	M	Single	Pakistan	2010	Tertiary	PhD researcher		English, Pakistani
Interview 3	23/10/2018	Early 40 s	M	Married with children	Sudan	2012	Tertiary	Student	Public Sector Official	English, Arabic
Interview 4	27/10/2018	Mid 30 s	F	Married with children	Iran	2009	Tertiary		n.a	English, Persian
Interview 5	27/10/2018	Early 40 s	M	Single	Iran	2011	Tertiary	Student and waiter	Civil Servant	English, Persian
Interview 6	28/10/2018	40 s	M	Single	Iran	2011	Tertiary	Engineer	n.a	English, Persian
Interview 7	01/11/2018	30 s	F	Married with children	Pakistan	2002	Tertiary	Not working	n.a	English, Pakistani
Focus Group	05/02/2019	70 s (8 WOMEN)	F	Married with children	India	1980	Primary	Retired	n.a	English, Indi
Interview 8	29/10/2018	30 s	F	n.a	Eritrea	2008	Tertiary	Policy officer	n.a	English, Arabic
Interview 9	20/06/2019	30 s	M	n.a	Nigeria	2008	Tertiary	Civil servant	n.a	English
Interview 10	1/07/2019	30 s	F	Married	Malaysia	2011	Tertiary	PhD student	Banker	English, Malay
Interview 11	1/07/2019	30 s	F	Single	Turkey	2006	Tertiary	PhD student	Secretary	English, Turkish
Interview 12	10/07/2019	40 s	M	Married with children	Salvador	2019	Tertiary	Not working	Lawyer	English, Spanish
Interview 13	8/07/2019	30 s	F	Married with children	Algeria	2015	Tertiary	Not working	Customer Support	English, Arabic, French
Interview 14	13/07/2019	30 s	M	Single	Nigeria	2013	Tertiary	System engineering	Computer engineering	English
Interview 15	13/07/2019	20 s	M	Single	Ivory Coast	2013	Tertiary	Bachelor student, waiter	n.a	English and French
Interview 16	18/07/2019	30 s	M	Single	Iraq/Iran	n.a	Tertiary	Not working	n.a	English, Farsi and Kurdish
Interview 17	22/07/2019	30 s	F	Married	South Africa	2018	Tertiary	studying	n.a	English
Interview 18	22/07/2019	30 s	M	Married	South Africa	2018	Tertiary	Mechanical engineer	Mechanical engineer	English

The TSOs involved in our study were selected based upon the variety of their activities and their prominence in providing services related to employability. Some organisations supported holistic integration services (such as information and guidance relating to welfare as well as English language classes) aiming at developing a positive environment which can enhance the employability of migrants, refugees and asylum seekers, while other organisations instead provided employability, volunteering or business support schemes, focusing directly on labour market integration. A third category of organisations was formed by work integration social enterprises that include migrants, refugees and asylum seekers in their workforce, as well as some non-governmental organisations mainly involved in implementing policy advocacy activities.

Migrants, refugees and asylum seekers interviewed presented very different pathways of migration, including economic migrants who have moved to the UK with the idea of establishing their life in the UK,, refugees who have obtained their status through the asylum process, refugees who have been resettled through one of the resettlement programmes and asylum seekers who are still waiting for their cases to be addressed. We have opted to include these 3 categories of newcomers because although they have different rights and different challenges when accessing employment, they are all encompassed within a political narrative of “deservingness”, a consistent policy discourse in the UK according to which they all must prove their “worth” to live in the country.

The majority of the interviews and focus groups were recorded and transcribed ‘intelligent verbatim’ and when it was not possible to record, extensive notes were collected by researchers. The confidentiality and anonymity of each interviewee were protected throughout the interview process. In doing so, interviewee numbers and roles (MRAs or TSOs managers) are used in detailing the quotes presented in this paper. Ethical approval was requested and obtained from the ethical committee of the University. The interviews were transcribed and the data were then imported into the computer-assisted qualitative data analysis software QSR Nvivo for two cycles of coding. An inductive process was employed. Two rounds of thematic coding were used for identifying the different themes analysed and to group concepts together (Miles and Huberman 1994; Saldana 2015) with a view to establishing the role of TSOs in facilitating the integration of MRAs into the UK labour market.

## Findings

In our findings, we are going first to provide an overview of the TTSOs dealing with integration in the labour market. Second, we are going to focus on the role that TSOs have in favouring (or not) employability of migrants, refugees and asylum seekers. We conclude than with an analysis of the effect of austerity on TSOs.

### Overview of TSOs Dealing with Integration of Migrants, Refugees and Asylum Seekers in the Labour Market

**Fig. 1:**
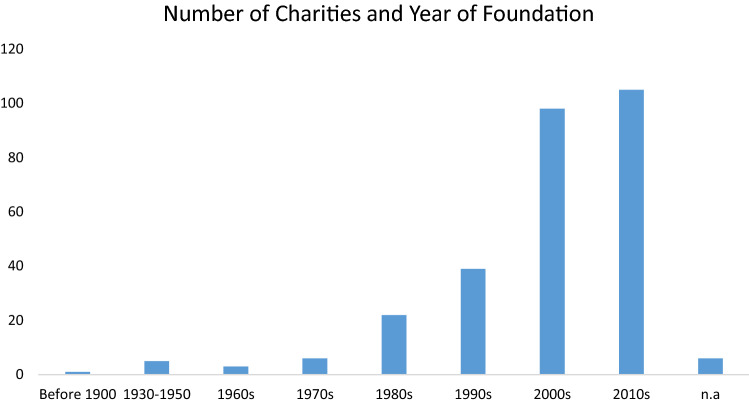
Number of Charities dealing with the employability of migrants, refugees and asylum seekers by Year of Foundation

A total of 285 organisations (out of 2,814,658 organisations registered in the databases) that focus on the labour market integration of migrants, refugees and asylum seekers have been identified. Figure 1 presents a breakdown of the number of new organisations across each decade since the beginning of the century. It reveals that 85% of the organisations which today are still active were created from the 1990s onwards, and 71% of the total number of organisations were founded between 2000 and 2019. This could be due to the increasing role, analysed earlier, that third sector organisations acquired in terms of service provision during the last three decades and by a policy paradigm that shifted the responsibility of increasing employability from employers and the government sector to the jobseekers and civil society sector (Montgomery et al. 2017, Jessop 1994, 2003). The increasing number of new TSOs focusing upon labour market integration in the UK does appear to indicate that there is a growing demand for services which focus on this specific aspect of integration. However, 98 organisations were founded between 2000 and 2009 while 105 were established between 2010 and 2019. Therefore, the so-called refugee crisis of 2014–15 does not seem to have affected the establishment of new organisations focusing on labour market integration, while it most probably affected the number of organisations focusing upon a wider range of services and needs.

The Charity Commission and regulator data alongside our analysis of the TSOs websites reveal that the activities undertaken in relation to the labour market integration of migrants, refugees and asylum seekers in the UK can be structured across five different typologies:

1. Employment activities: this category includes not only services which provide a match or a bridge between companies and beneficiaries but also migrant-led organisations which employ migrants and refugees in their workforce and TSOs which support migrants and refugees to start-up businesses.
2. Integration support activities: this category mainly includes organisations which provide a holistic integration service in which employment is aligned with advice-based programmes concerning housing, welfare benefits or health.
3. Education and training activities: this category includes mainly those organisations which organise English language courses.
4. Skills development activities: this category includes mainly those organisations which provide courses for developing skills related to specific jobs.
5. Policy advocacy activities: this category includes mainly organisations which advocate for the equality of migrants in the workplace or promote campaigns related to the rights of migrants, refugees and asylum seekers employed in the UK.

As presented in Table 3, the majority of organisations in our dataset are providing integration support services (38%) and training and education services (30%). Examining our findings more closely we can observe that 14% of TSOs promote policy advocacy related to employability while only 13% provide employability services and 4% invests in skills development activities. From this perspective, it is clear that the main activities that TSOs undertake are related to language provision on the one hand and a holistic approach to integration on the other hand. Employment services and skills development activities seem to be only residual in terms of the range of activities that TSOs provide and they are often aligned with other types of services.

**Table 3:** TSOs’ Area of activities (Percentages)

Area of Activities	Percentages
Integration support	38%
Training and education	30%
Policy advocacy	14%
Employment	13%
Skills development	4%
Other	1%
Total N	388

We can conclude that a limited number of organisations provide formal employability services or skills development services, while most focus upon education and training activities such as the provision of English language courses. Perhaps this reflects the marketisation and privatisation trend that we have presented earlier and echoes the findings of the Big Society audit (Kendall et al. 2018) showing that further opportunities have been provided and seized by larger private companies or social operators than charities and civil society-grassroots organisations. Nevertheless, this finding also reflects the fewer financial resources available to promote employability activities and the austerity measures which have particularly affected these TSOs, reducing the availability of services for migrants, refugees and asylum seekers. Another possible explanation is that employability services are not regarded by TSOs as the most important activities to provide for the effective integration of migrants, refugees and asylum seekers in society. They could instead be considered a secondary activity which can be provided by other organisations such as recruitment agencies and/or public sector agencies, once a first level of integration activities has been delivered.

## A Marginal but Fundamental Role in the Integration of Migrants, Refugees and Asylum Seekers in the UK Labour Market

The marginal role in providing services for employability identified through our categorisation was in some part confirmed by our qualitative analysis. However, we noticed that TSOs provided a somewhat different mechanism of labour market integration according to the different type of migrant and migration rationale.

### Economic Migrants Experiences of TSOs Services

Among the interviews we conducted with “economic migrants”, that is those who entered the UK mainly through the tertiary education system (to enrol in Masters or PhD programmes) and who remained to find work, there was no knowledge nor experience about the services that TSOs offer in relation to employability and accessibility to the job market. Economic migrants mainly used either university career services or, as elaborated in the extant literature (Ambrosini 2016) relied upon help from friends with more experience, whereas others went on to “conduct research on how to apply for jobs and conduct interviews on the internet” (14_MRA).

A support network of people and opportunities to gain work experience in the UK context were instead pinpointed by all interviewed migrants as the main enablers to access the job market and it is through these two avenues that TSOs have a key role to play in labour market integration. Although migrants did not use the services provided by TSOs, they indirectly encountered these when using organisations’ volunteering schemes and their participation in community-based or faith-based organisations. Volunteering was often used to expand the social network of people and provide work experience in the UK which was perceived as often more appreciated by prospective British employers than work experience acquired abroad: “volunteering was useful in different ways. My network improved. Positive well-being to know other people increased, at a time that I was struggling to get a job, it helped. Here they don’t care about education as much as they care about experience. Even though I worked as a secretary in Kuwait that somehow didn’t translate as experience here, while working in a charity shop translates more because it was in the UK but I had more responsibility as a secretary in Kuwait” (11_MRA).

Participation instead in community groups, such as faith organisations and specific BAME groups, helped to develop a sense of community and integration, promoting trust and confidence alongside widening the network of people. As suggested by the focus group we conducted, their community-based organisation “started with the idea of promoting the role of women in society, promoting the appreciation of arts and culture” and eventually they became a point of reference for all the women of that community, widening job opportunities and developing new relationships that benefit both them and wider society.

### Refugees and Asylum Seekers Experiences of TSOs Services

Contrary to economic migrants’ experiences, all refugees and asylum seekers had some direct experience of interaction with TSOs which were identified by our interviewees as providing a first level of support and integration services. They were in fact pinpointed as providing help with asylum cases, the provision of basic needs such as food and clothing, advice about housing and benefits and providing English language classes in the community. As suggested by one interviewee, “when you arrive in the UK as asylum seekers you have priorities. First to get the status. Second is housing” and “TSOs help you in an emergency, school, housing, benefits, GPs” (3_MRA). However, as suggested by another of our interviewees, the lack of emotional support alongside difficulties in accessing training and education are often experienced by refugees and asylum seekers. Only when they discover the support that is offered by TSOs, sometimes through their networks of families and friends, can they get a “bit more financial and educational support” (16_MRA). Only afterwards and usually after several years, is a second level of integration provided, which mainly involves language classes, volunteering schemes and eventually employability placements. However, reflecting the views of a number of our interviewees, one of our participants explained that “it is not part of the duties of non-profit organisations to find a job” (3_MRA_UK) but it is up to the “personal life” (5_MRA_UK) to choose what to study and where to work and to build up the pathway to achieve personal aspirations.

English language classes and volunteering schemes were identified by interviewees as the 2 key activities provided by TSOs more connected to the UK job market. Interviewees suggested that English classes provided in the community were the first point of contact for starting to learn the language. For example, 2 interviewees were referred (through a social worker or friends) to English language classes in the community while they were waiting to access a further education college for a more formal learning programme. Long waiting lists to access English language classes in colleges as also reported by extant research (Meer et al. 2019) affected the lives of people and the possibility of acquiring a higher level of language proficiency more quickly; language classes in the community were identified as a temporary solution “that is better than nothing” (16_MRA) although limitations were also identified. Often the lack of resources of TSOs was identified as barriers to effective language courses: the availability of only one teacher and one group of beneficiaries with very different levels of English proficiency or the lack of IT equipment were perceived as reducing the effectiveness of the classes and the opportunity to acquire a good standard of English.

Volunteering instead within TSOs was recognised as particularly important to widen a person’s network of acquaintances, increase confidence and trust and finding new job opportunities. TSOs were perceived as a safe space to meet new people and build up the confidence which is often important for accessing jobs in the future, alongside having the opportunity of undertaking work experience in the UK. Finally, among our interviewees, only one person used an employability service provided by a TSO. Thanks to the service, he undertook two placements and he was offered a job. However, he chose to continue studying at college which would furnish him with a higher qualification and he felt that it was important to understand the different culture of the UK and acquire more confidence before undertaking an employed position.

Although TSOs provide first level integration services and try to address as best as they can at least the most urgent and pressing needs in particular of refugees and asylum seekers amidst a hostile policy environment, two limitations were singled out by our interviewees.

First, it was perceived that if you were not identified as belonging to a specific pathway of migration, whether you were an asylum seeker, refugee or migrant, then it is difficult to access any kind of support service. As suggested by one interviewee who was a migrant himself and the founder of a TSO, the first question while he was asking for support was “are you in this country illegally, no; are you an asylum seeker, no; are you a refugee, no; are you an international student, no. Sorry we can't help and for us that experience taught us to get to a place where we certainly decided if we ever set up something first and foremost we have that relationship but secondly, we don't want to form an organisation that is going to kick out anyone” (5_TSO_UK). Therefore, most probably due to the difficulties they are experiencing in terms of resources, organisations may risk unwittingly excluding vulnerable people who for different reasons are falling “between the cracks” because these individuals are not easily identifiable within one or more category for whom services are provided. Second, a lack of coordination among TSOs was also identified by our interviewees. As suggested by another TSO interviewee, although these organisations really want to help, there is often a lack of coordination and a lack of communication: “some people don’t know any of these organisations, so if you don’t know any, it is difficult. I also think that asylum seekers, organisations don’t engage with them because they are not allowed to work, so they just leave them in the ESOL classes” (8_TSO_UK). Thus, if migrants, refugees and asylum seekers are better informed about the existence of these organisations, it most probably means that they have already established a network of people or organisations which can support them or they live in an area where the support is more easily available.

## The Effect of Austerity on the Response Capacities of Third Sector Organisations

Our findings confirm that the reduction of public funding has necessarily impacted the number of organisations which provide public sector type provision. As suggested by one interviewee, “there were other agencies we used to refer to, they have been downsized very much; they don’t have much resources. They do some schemes for refugees for doctors, they run a few activities, but it is nothing compared to what was run before. Some organisations have closed. There is less infrastructure to help people from our country find jobs” (13_TSO). These concerns were echoed by another of our participants, “Home Office funding for refugee integration employment services was withdrawn” and the “entire refugee sector was shut down” (4_TSO_UK). This affected not only community organisations which had to continue, in the best case scenario, to provide more services with the same amount of resources but also more established TSOs which had to completely restructure their integration services, reducing for example employment schemes and services. Accessibility to public funding was also identified as particularly difficult due to the specificity of contracting mechanisms. The founder of one of the TSOs participating in our study described the challenge of his organisation “to even get a public contract because of the systems in place” (5_TSO_UK) which seemed to favour larger and more established organisations that have more structured procedures and present a lower level of risk for government commissioners.

The TSOs resourced by the Home Office mainly provide information and advice for migrants, refugees and asylum seekers, while in recent years few employability schemes and pilot programmes have been resourced by the UK Government. Providing services for the UK Government does not come without tension, particularly if, for example, the lack of participation by MRAs in some of the workshops or services provided by TSOs can result in the sanctioning of beneficiaries and reducing their benefits and rights. Thus, as suggested by one organisation we spoke to, TSOs should be “aware of the risks of working with the UK Government and to be very clear about the conditions connected to the programmes” (7_TSO_UK).

Less tension was instead identified in the provision of services for the Scottish Government or the local authority resettlement schemes, which have a remit of integration but have no responsibility for border control. For example, as stated by one interviewee in Scotland, the decision they took to include in their programme—which previously only focused on refugees and asylum seekers, migrants—third country nationals and EU nationals, was a consequence of a request by the Scottish Government to expand the pool of beneficiaries. However, at the moment due to the political instability that is characterising the UK context and the approaching end of the resettlement programme,^2^ it is difficult for TSOs to understand if in the future “they will focus so much on integration or they will need to do a lot more on the policy and advocacy side” (2_TSO) because of the potential consequences of policy change at the UK Government level.

Almost all of the organisations we interviewed advocated for improvement of policy through different instruments. Some of them explained that they often participate in consultations at the UK Government level such as one related to the future of the resettlement programme after 2020 or they work in collaboration with specific All Party Groups or commissions to improve Home Office procedures and future policies. One interviewee pinpointed that the type of advocacy they do can best be described as “collaborative influencing”. They perceived themselves to be collaborative, aiming at improving the system without however taking any political stance, which could negatively impact their existing relationships with other stakeholders. However, some organisations declared that although they are involved in different consultation events with a collaborative lens, they recognise that their contribution is not taken into consideration: “So within that discussion, you contribute and participate and all that, and you realise that your contribution is not valid. So yes, they give you the power to make the decision and to be engaged… but the recognition is not there, and it goes back to that point of just ticking the box” (5_TSO). Other organisations instead took a more po*l*itical stance, aimed at stimulating change in the system through campaigning (e.g. the ‘Lift the Ban’ campaign to allow asylum seekers access to work or the ‘Stand as one’ campaign to support the integration of refugees) or through the organisation of events (e.g. Day of Action). More established organisations tried to use their profile to access politicians and policy makers and give a voice to people that usually struggle to be included in policy dialogues. One TSO, for example, organised what they called “candidate cafes” which involved organising events across different communities, inviting all candidates for the local election alongside community groups to discuss future policies together.

## Discussion

Reflecting upon our analysis, three main considerations can be drawn. The first consideration relates to the positive (primarily social) effects that TSOs can bring to the field of labour market integration for migrants, refugees and asylum seekers. The remaining 2 considerations shed light on the environment (particularly the economic and political contours of the UK context) in which these organisations have been operating and which raise ongoing challenges.

Firstly, consistent with earlier studies we found that it was primarily refugees and asylum seekers that have been involved in using services and activities provided by TSOs (see for example Mayblin and James 2019), while so-called economic migrants tend to use their more informal networks, career services and recruitment agencies to gain access to the UK labour market. Economic migrants use third sector organisations to become more involved in the community where they live, through for example, volunteering. Through TSOs, they also build their informal networks and work experience which are essential to gain access to the labour market. For refugees and asylum seekers, instead TSOs are the main providers of integration support services and training education services (Mayblin and James 2019). Moreover, they represent the main source of referral to various organisations in the public, private and elsewhere in the third sector and thus play a crucial actor in signposting refugees and asylum seekers to those services that can help them address their most pressing and urgent needs (Strokosch and Osborne 2016). Only a limited number of organisations instead provide formal employability services or skills development services which seem to be only residual in terms of the range of activities that TSOs can organise. Partially this derives from the fact that employability services are not regarded by TSOs as the most important activities to provide for the effective integration of migrants, refugees and asylum seekers into society. They instead appear to be considered as a secondary activity best provided by other organisations such as recruitment agencies and/or public sector agencies, once the most urgent needs have been addressed. Nevertheless, volunteering schemes have been highlighted by migrants, refugees and asylum seekers themselves as one of the most important activities related to TSOs and for labour market integration (De Jong 2019). TSOs provide a space where people with different pathways of migration can widen their social networks and obtain some form of work experience in the UK labour market, experience that is perceived as being of particular importance to potential employers who are understood, from the perspective of our interviewees, to be less responsive to the value of work experience gained prior to migrating. Therefore, when we consider the impact of TSOs on the labour market integration of migrants, refugees and asylum seekers, it may be tempting to focus on the fact that this seems to be of secondary importance to the meeting of more pressing needs, particularly for the latter two groups. Instead, our findings perhaps uncover the positive and hitherto undervalued ‘spillover effects’ of the activities undertaken by TSOs to meet pressing needs on the labour market integration of migrants, refugees and asylum seekers. These effects are manifested through the provision of a safe space for service users that allows them to increase their confidence in integration activities such as learning the language in an environment that recognises the specific challenges of their migration/asylum journey (De Jong 2019). These effects also include the building of social capital which not only assists in improving the well-being of service users but also provides new points of connection in their host communities (Putnam 2004). These connections can expand their horizons in terms of educational and employment opportunities as well as facilitate their full participation in society and in spaces that can be considered less safe (Granovetter 1977, 1983; Barbieri 2000). Finally, in a more direct way, these effects are manifested through opportunities for volunteering that can bolster the CVs of those involved with work experience that is valued by local employers.

Turning next to a key feature of the environment that TSOs are navigating in the UK, the austerity measures throughout the last decade have particularly affected TSOs which work with MRAs, reducing the availability of services (and specifically those in the field of employability) for migrants, refugees and asylum seekers. Not only have austerity measures impacted upon potential partners in local government more acutely (Lowndes and McCaughie 2013) but they have also affected the relationship between the UK Government and TSOs. The commissioning procedures raise barriers for the involvement of TSOs, favouring private for-profit companies or larger and well-established organisations instead of smaller, grassroots oriented organisations (Egdell et al. 2016). Providing services for the UK Government (such as the Home Office department) does not come without tension and the reputational risk of receiving funds or negotiating objectives with a UK Government which has consistently demonstrated its concern for border control rather than integration (Walters 2004; Squire 2017).

In response to the absence of funding from the UK Government, each of the TSOs we interviewed were seeking alternative sources of income. However, resources scattered across different funders affected not only the long-term sustainability of TSOs but also their capacity to tailor their services, pursue their social mission and realise their goals to assist those people who are among the hardest to reach (Lyndsay et al. 2014; Shutes, 2011). For example, without adequate resources it is the most vulnerable people and among them asylum seekers that risk being inadvertently excluded from the different types of support TSOs can offer (Shutes 2011). It is clear from our findings that if these people do not have access to such services, it may become more difficult in the future to support their integration into the labour market and thus they risk remaining on the margins of UK society.

On a related point, the organisations participating in our study would tend to become competitors struggling over a shrinking pool of funding and were therefore embedded in a continuous cycle of short-term projects rather than pursuing synergic relationships and long-term goals (Zimmerman et al. 2014). Therefore, funding challenges can fundamentally jeopardise the activities of TSOs and represent the main barrier hindering their work to promote the integration of migrants, refugees and asylum seekers into the UK job market.

Another important feature of the environment which TSOs in the UK must navigate is the political context in which the contentiousness surrounding migration has become intensified, further reducing the willingness of the UK Government to fund organisations working in this field (Dennison and Geddes 2018). Although, a clear divergence in terms of the narratives and policies between Westminster and Holyrood is evident from our analysis (see for example the New Scots Refugee integration strategy 2018–2022), the lack of long-term financial investment in employability programmes could jeopardise the effectives of the positive rhetoric and narratives that have shaped the direction of travel of the Scottish Government. We found that TSOs mainly occupy a space of collaborative influencing (aiming at improving the system without however taking any political stance) more than service delivery or partners in designing policy and promoting new services (the exception to this could be the New Scots Refugee Integration Strategy promoted by the Scottish Government, which potentially offers a space for more effective forms of partnership, although it is still at an early phase of implementation), and thus have limited impact on policy change.

Although almost all of the organisations we interviewed advocated for an improvement of policy through different instruments, there is the perception of a lack of visibility and recognition by policy makers, which seem to mobilise the involvement of TSOs as a tick-box exercise rather than a valuable and informative contribution. The forms of co-governance and co-management identified by Strokosch and Osborne (2016) in Scotland were not identified at the national level, where most probably a more marketised agenda is promoted. On the other hand, the organisations that take a more political stance, campaigning for more systemic change seem to have better access to media platforms and contribute to widening information sharing about the topic of integration into the labour market in particular the most pressing issues facing refugees and asylum seekers. However, it is not yet possible to assess the impact of these campaigns on changing policies and discourses or whether they increase the visibility and recognition of TSOs to policy makers.

## Conclusion

Our findings illustrate how budget cuts in social policy programmes evidenced in the extant third sector and social policy literature (see for example Kendall et al. 2018, Alcock 2016) reflects a weaker capacity of societal action in a sector such as labour migration when there is so much emphasis placed on the capacity of migrants to integrate by means of finding a job. What we argue is that regardless of the goodwill of newcomers, the services provided by third sector organisations (TSOs) in labour migration integration are limited. Nevertheless, they still serve as important vehicles of integration especially for the most vulnerable groups (refugees and asylum seekers) by offering opportunities for socialisation and networking, as well as gaining work experience via volunteering. Furthermore, they represent the main source of referrals to different organisations and are often a key hub for refugees and asylum seekers, to address their most pressing and urgent needs. Our findings also reveal that TSOs provide a safe and trusted environment that people can use, according to their experiences and pathways of migration, to increase their confidence, improve their well-being, to broaden their social circle, to learn the language or to increase their work experience. Therefore, although from a narrow perspective we may say that TSOs perform a rather limited direct role (in terms of service provision) in the area of labour market integration and thus may well be ‘marginal players’, adopting a broader lens of analysis allows us to appreciate those efforts to meet more basic needs for integration that can involve the accumulation of various ‘spillover effects’ (of networks, better information as well as opportunities for work experience) that can provide a foundation for more successful outcomes in labour market integration. Practitioners should thus continue to provide services which indirectly through work experience and rising social capital can contribute to the integration of migrants, refugees and asylum seekers into the labour market. Policy makers should also recognise the role of third sector organisations, providing long-term investment which can be used to assist these organisations in this role.

Regarding the limitations of our study, we are aware that our selection and categorisation of TSOs underestimates the level of support provided by TSOs to migrants, refugees and asylum seekers. For example, some organisations are not required to register with the Commission (such as small community-based organisations with an annual income of under £5000). For other TSOs it was difficult to explore if they were undertaking activities related to employability services. Since our study has focused on the UK only, it would be difficult to generalise our findings to other contexts. Future studies should explore the role of TSOs in countries in which the relation between TSOs and the public sector have been shaped by different policies. In addition, exploring for whom, in what context and how third sector organisations might affect employability of migrants can provide further evidence (qualitatively and quantitatively) of the role of third sector in this contested field of policy.

## Footnotes

1 A categorization of third sector organisations (TSOs) dealing with refugees and asylum seekers in the UK (see Mayblin and James 2019) has been recently conducted. The categorisation conducted by Mayblin and James, (2019) focus upon all integration services provided to refugees and asylum seekers in England and Wales, excluding organisations that deal with migrants or BAME communities and not taking in consideration third sector organisations in Scotland and Northern Ireland.

2 The resettlment programme had been confirmed up to 2020 but was then suspended in March 2020 due to the covid-19 epidemic.
